# Editorial: Effects of midday naps on glycemic control of diabetic patients

**DOI:** 10.3389/fendo.2024.1430924

**Published:** 2024-05-21

**Authors:** Shinya Makino, Mohammed A. Al-Abri

**Affiliations:** ^1^ Department of Internal Medicine, Osaka Gyomeikan Hospital, Osaka, Japan; ^2^ Department of Physiology, College of Medicine and Health Sciences, Sultan Qaboos University, Muscat, Oman

**Keywords:** midday naps, nighttime sleep, napping type, glycemic control, diabetes

Sleep is an important component of daily life in modern societies for maintenance of good health status. A growing body of evidence suggests that short and/or long nighttime sleep duration increases the risk of diabetes and aggravates the glycemic control in diabetic patients (1, 2). Because midday naps could modify such associations, the researchers have been paying much more attention to napping behavior than ever. While it has been consistently reported that midday naps, especially if it is long (>1h), could be associated with higher risk of diabetes (3, 4), previous findings about influence of midday naps on glycemic control are conflicting (5).

Nevertheless, we found that taking midday naps reduced the risk of poor glycemic control of short nighttime sleepers (<5h/night) with type 2 diabetes who complained of low sleep satisfaction and daytime sleepiness (6). The results were striking and suggests that midday naps might be specifically beneficial to short sleepers who need to take midday naps in response to sleep loss (replacement napping) or in preparation of sleep loss (prophylactic napping). However, the small sample size in our study made us to decide to set up this Research Topic which focus on whether the effects of midday naps on glycemic control depend on type of napping as well as on nap duration.

Kakutani-Hatayama et al. examined insulin secretion and sensitivity in 436 diabetic patients. Decreased insulin sensitivity was observed in long (>1h) nap group, but not in short (<1h) nap group. Furthermore, an association between decreased insulin sensitivity and long naps was more prominent in short nighttime sleeper (<6h) than in subjects with longer nighttime sleep (>6h). Their findings are consistent with a previous report showing that long (>1h) midday naps was a risk of diabetes development irrespective of nighttime sleep duration, but had the higher risk for individuals who slept <5h at night (7).

Yao et al. reported that poor nighttime sleep quality is independently associated with increased risk of hypertension, whereas short midday naps (1-30min) was negatively correlated with the risk of hypertension in 1722 patients with type 2 diabetes. Although they did not find significant difference in glycemic control among participants with different sleep parameters (nighttime sleep duration or midday nap duration), they suggested beneficial effect of short napping on the risk of hypertension in type 2 diabetes.

Yuan et al. examined the association between napping characteristics (type, frequency, duration and timing) and glycemic control in 226 participants with type 2 diabetes. This epoch-making article fits the scope of our Research Topic very well. Napping type was divided into two categories; i.e. appetitive (for enjoyment) and restorative (replacement and prophylactic) napping. They found that long napping duration (>60min) and morning napping timing were associated with poor glycemic control. Furthermore, restorative napping was related to better glycemic control as compared with appetitive napping. To the best of our knowledge, this is the first study to demonstrate the relationship between type of napping and glycemic control.

During the period of the Research Topic, one review article on the relationship between napping and type 2 diabetes was published in this journal (8). Long naps (>30-60min) induce sleep inertia, circadian rhythm disruption and nocturnal sleep disturbance, which stimulate the production of proinflammatory cytokines, increase afternoon cortisol levels, and decrease melatonin secretion. All these changes are associated with insulin resistance and obesity, resulting in increased risk of type 2 diabetes. However, beneficial effects of short naps may depend on the nighttime sleep quantity/quality. If nighttime sleep is sufficient or even excessive, napping is not recommended or should be restricted to 30min. In contrast, appropriate napping (<30min) may be needed to compensate for insufficient nighttime sleep (9). We think that this appropriate napping should be restorative napping.

Overall, although our Research Topic proposed a new approach for napping research, evidence for the specific role of restorative napping on glycemic control are still lacking. We are just at the beginning of the new stage of napping research. Much more studies from the wide range of areas, communities and races are definitively necessary to determine how we should intervene in sleep-related behavior including midday naps for better glycemic control in type 2 diabetes. We need to solve the problem “to nap or not to nap for the restorative reasons in diabetic patients with short/insufficient nighttime sleep?”.

## Author contributions

SM: Writing – original draft, Writing – review & editing. MA: Writing – review & editing.
